# Mechanical and Corrosion Properties of Mg-Based Alloys with Gd Addition

**DOI:** 10.3390/ma12111775

**Published:** 2019-05-31

**Authors:** Aneta Kania, Ryszard Nowosielski, Agnieszka Gawlas-Mucha, Rafał Babilas

**Affiliations:** Institute of Engineering Materials and Biomaterials, Faculty of Mechanical Engineering, Silesian University of Technology, Konarskiego 18a, 44−100 Gliwice, Poland; ryszard.nowosielski@polsl.pl (R.N.); agnieszka.gawlas04@gmail.com (A.G.-M.)

**Keywords:** Mg-based alloys, microstructure characterization, mechanical properties, electrochemical testing, immersion tests

## Abstract

Magnesium alloys with rare earth metals are very attractive materials for medical application because of satisfactory mechanical properties. Nevertheless, low corrosion resistance is an obstacle in the use of Mg alloys as resorbable orthopedic implants. The paper presents results of mechanical and corrosion properties of MgCa5-xZn1Gdx (x = 1, 2, and 3 wt. %) alloys. Based on the microscopic observations it was stated that the studied alloys show a dendritic microstructure with interdendritic solute rich regions. The phase analysis reveals an occurrence of α-Mg and Mg_2_Ca, Ca_2_Mg_6_Zn_3_ phases that are thermodynamic predictions, and stated Mg_26_Zn_59_Gd_7_ phases in MgCa5-xZn1Gdx (x = 1, 2, and 3 wt. %) alloys. The Mg_26_Zn_59_Gd_7_ phases are visible as lamellar precipitations along interdendritic regions. It was confirmed that an increase of Gd content from 1 to 3 wt. % improves ultimate tensile (R_m_; from 74 to 89 MPa) and compressive strength (R_c_; from 184 to 221 MPa). Moreover, the studied alloys are active in Ringer’s solution. They are characterized by an increase of corrosion potential (E_corr_) of about 150 mV in comparison with values of open circuit potential (E_OCP_). The best electrochemical parameters (e.g., corrosion current density, i_corr_, polarization resistance, R_p_, and E_corr_) were obtained for the MgCa3Zn1Gd2 alloy.

## 1. Introduction

The scientific literature contains many publications on the use of magnesium, calcium, and their alloys as resorbable materials for medical purposes [1,2,3,4,5,6,7,8,9,10]. The materials used for biomedical implants should be characterized by an appropriate load bearing capacity, especially in the case of small implants, biocompatibility, good mechanical properties, lack of clotting tendencies, even the ability to recycle, and high corrosion resistance in the tissue environment [10,11,12,13,14,15,16,17]. It is not possible to design the perfect alloy that connects all the required parameters. The problem is the poor corrosion resistance of Ca and Mg alloys [4,8,18]. In this respect, it should be noted that the environmental influence on corrosion affects the application of Mg alloys [19]. The corrosion rate is slow, for example, in pure water, but it is significant in chloride solutions (e.g., Ringer’s solution that simulates the physiological environment) and acid solutions [19,20,21]. Knowing and understanding magnesium corrosion rate in vivo is an important issue for an application of Mg alloys in medicine [22,23,24,25,26,27,28]. To understand the corrosion mechanism of magnesium alloys, it is important to know the corrosion mechanism of pure Mg [19,20,29]. It is also necessary to take account of the influence of metallurgical factors on corrosion of Mg-based alloys (e.g., the reactivity of Mg, the presence of second phases, the influence of surface films and the influence of alloying elements on corrosion protection) [19,23,24,30,31,32,33,34,35]. Some elements have a detrimental influence on the corrosion of Mg alloys. The impurities (Ni, Cu, Co, and Fe) significantly accelerated the corrosion of binary Mg alloys [25]. Thus, it is important to keep the tolerance limit of the impurity concentration [20,21,23,25]. The proper selection of alloying elements is a fundamental matter for resorbable alloys design. They have an essential influence on corrosion mechanism, especially in the case of films formation and hydrogen evolution [20]. The compounds formed during implant dissolution must not be toxic to the human body [5,10,11,12,36]. It is also important to know the correlation between the dissolution rate of the material [20] and its mechanical properties. Too fast of a degradation process of Mg-based implants can lead to the lack of implant integrity with a tissue and a lack of adequate implant products that accumulate too fast around the implant and consequently cause an inflammation in the human body.

Recently, some results of microstructure and mechanical property investigations of Gd-containing alloys have been reported [12,13,14,15,18,30,31,32,33,34,37,38,39]. Liu et al. [37] examined the Mg-4.58Zn-2.6Gd-0.18Zr alloy after different heat treatments. The as-cast alloy was composed of primary α-Mg, (α-Mg + Mg_3_Zn_3_Gd_2_) eutectic, Mg_3_Zn_6_Gd phase, and Mg_3_Gd particles within α-Mg. They stated that after a solution treatment at 505 °C for 16 h and an aging treatment at 220 °C over 16 h, changes in the microstructure of the alloy resulted in the improvement of mechanical properties. The microstructural evolution, phase constitution, and mechanical properties of Mg-5.5Zn-xGd (x = 0.8, 2, and 4 wt. %) alloys after directional solidification were studied by Yang et al. [38]. They confirmed that an increase of Gd content from 0 to 2 wt. % caused the increase of tensile strength (R_m_). Above 2 wt. % of gadolinium the R_m_ decreased. There is no doubt that microstructural parameters (e.g., grain size or phase distribution) and crystallographic orientation are significant in determining the corrosion behaviors of Mg alloys [21,40].

The article presents the results of microstructure, mechanical properties, and corrosion resistance investigations of quaternary MgCa5-xZn1Gdx (x = 1, 2, and 3 wt. %) alloys as potential candidates for orthopedic implants. The composition of studied alloys was designed due to high solid solution strengthening of Mg alloys with Gd [14,32,34] and the results were also presented by Zheng et al. [17]. The minor Gd addition (1–3 wt. %) in Mg-based alloys was dictated by an increase of ultimate tensile strength and decrease of degradation rate. The authors [17] stated that the degradation rate decreased up to 7 wt. % of gadolinium. Therefore, taking into consideration the above results, the authors intended to verify the effect of minor gadolinium addition (from 1 to 3 wt. %) into Mg-based alloys on the improvement of mechanical properties and corrosion resistance.

Implants prepared from magnesium alloys with calcium and zinc after implantation in the human body slowly undergo spontaneous resorption. The addition of Ca into Mg-based alloys causes the increase of their strength, hardness, and resorbability [32]. Zinc improves tensile strength and hardness. This element increases the tolerance limits of impurities in Mg alloys [21]. On the other hand, the addition of gadolinium improves the strength properties and reduces the effect of microporosity resulting from the shrinkage of magnesium alloys [34]. Besides the strength and ductility improvement of Mg alloys, Gd addition also improves the corrosion resistance. Magnesium, calcium, and zinc are biocompatible materials. In the case of gadolinium, there are not so many reports on its biocompatibility. It is known that Gd is characterized by a high solubility in solid Mg at eutectic temperatures [17], because of that it influences the reduction of intermetallic phases distribution and galvanic coupling formation. Therefore, Mg alloys with Gd addition are selected for biomedical alloys. Recently, some investigations of in vivo and in vitro studies with Gd-containing Mg alloys were carried out. Myrissa et al. [41] performed an in vitro qualitative analysis of cell distribution and viability of the binary Mg10Gd alloy. They stated that after 13 days of culture in a proliferative medium the viable cells on the studied alloy were revealed. Gadolinium added into Mg-based alloys may be a promising material to use in medicine, but there is a need for more in vivo and in vitro studies as well as clinical investigations.

The aim of the study is to investigate the hardness, tensile, and compressive properties of the alloys with minor addition of gadolinium (from 1 to 3 wt. %) at the expense of decreasing calcium content. Moreover, the evaluation of corrosion resistance of the Mg-based alloys with Gd is also a complementary purpose of the investigations.

## 2. Materials and Methods

The research materials included three magnesium-based alloys in as-cast state: MgCa4Zn1Gd1, MgCa3Zn1Gd2, and MgCa2Zn1Gd3. 

Mg-based alloys were prepared by induction melting in an argon atmosphere with technical purity (99.9%). Pure magnesium (99.99%) in the form of ingots, zinc in the form of rods (99.99% pure), calcium (99.5% pure), and gadolinium granules (99.9% pure) were melted in chamotte–graphite crucibles. The molten ingots were homogenized by annealing over 30 min at the temperature of 750 °C. The molten alloys were cast into sand molds hardened with a polymer (FIBRAL). The obtained plates had dimensions of 100 mm × 280 mm × 14 mm.

### 2.1. Structure Testing and Phase Analysis

The microstructure of studied alloys was observed on transverse microsections in the Zeiss SUPRA 35 scanning electron microscope (SEM, Thornwood, New York, NY, USA); extra-high tension (EHT) = 20.00 kV; working distance (WD) = 10.5 mm, backscattered electrons (BSE) mode equipped with an energy-dispersive X-ray spectrometer (EDS).

The microstructure was also observed by a high-resolution transmission electron microscope S/TEM TITAN 80-300 FEI (Hillsboro, OR, USA) equipped with a Cs-corrector. The investigations with an acceleration voltage of 300 kV were performed. The high-resolution transmission electron microscope (HRTEM) images and selected area electron diffraction (SAED) patterns were collected. Scanning transmission electron microscope (STEM) imaging was performed using a high-angle annular dark field (HAADF) detector that allowed us to distinguish a difference in the value as a result of the difference in scattering. Electron beam with a convergence semi-angle of 17 and 27 mrad was used. Samples for observations were prepared by gallium ion milling.

The phase analysis of the samples was carried out in the PANalytical X-ray diffractometer (X’Pert PRO model, Almelo, The Netherlands) using the Co Kα radiation (with a wavelength of 0.1789 nm). The measurements using the step registration method in the angular 2θ range from 30° to 90° were conducted. The X-ray qualitative analysis was performed using the HighScore Plus software (3.0e version, PANalytical, Almelo, The Netherlands).

### 2.2. Mechanical Tests

The tensile and compressive tests were carried out at room temperature with the ZWICK Z100 static material testing machine (Kennesaw, GE, USA) according to the standards [42,43]. The cylindrical samples with working diameters of 5 mm and lengths of 90 mm were prepared for tensile tests [42]. A pre-load force during the test was 2 N and the strain rate was 0.0067 s^−1^. The samples for static compression tests had diameters of 10 mm and heights of 15 mm [43]. A pre-load force of 2 N and beam move speed of 100 mm·min^−1^ were assumed. The Vickers hardness tests were performed with the Future-Tech FM-ARS 9000 device (Kawasaki, Kanagawa, Japan) under the load of 1 N. For each studied alloy 10 measurements were performed.

### 2.3. Electrochemical and Immersion Tests

The electrochemical and immersion tests were performed in Ringer’s solution at 37 °C. The Ringer’s solution simulates the physiological environment of a human body. The electrochemical tests were carried out using the Autolab PGSTAT302N Multi BA potentiostat (Herisau, Switzerland). The open circuit potential (E_OCP_) measurements and the potentiodynamic tests were carried out. The measurements were performed in a three-electrode cell with a water jacket using a saturated calomel electrode (SCE) as a reference electrode, a platinum rod as a counter electrode, and a sample as working electrode. The corrosion potential scan rate was 1 mV·s^−1^. In case of corrosion tests, changes of the open circuit potential as a function of 3600 s were collected. The exposure for the corrosion environment during immersion tests was 48 h. 

The cylindrical samples with a testing area of 1.1 cm^2^ were prepared for electrochemical tests. The orifice of the sample holder had a diameter of 10 mm. For immersion tests, rectangular samples with dimensions of 5.9 mm × 5.9 mm × 17.5 mm were prepared. The basic corrosion parameters of the studied alloys were determined using the Tafel’s analysis. The corrosion rate, v_corr_, based on the corrosion current density (i_corr_) value was also calculated. The hydrogen evolution volume was measured and calculated taking into account a surface of the samples. After immersion tests the corroded surface with corrosion products for each of the samples was observed by the SEM method; the investigation was carried out on gold sputtered samples. Additionally, the samples surface without corrosion products was observed using a Zeiss SteREO Discovery stereoscopic microscope (Thornwood, New York, NY, USA). The samples were rinsed with distilled water and immersed in CrO_3_ solution before microscopic observations.

## 3. Results and Discussion

**Figure 1:**
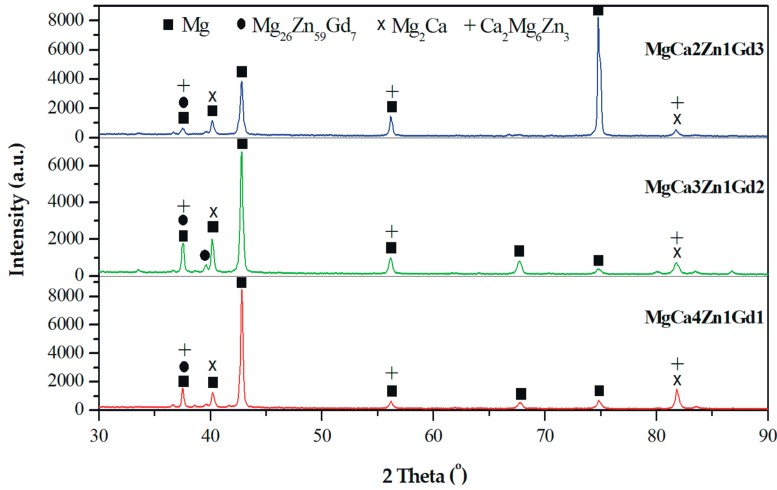
X-ray diffraction patterns of MgCa5-xZn1Gdx (x = 1, 2, and 3 wt. %) alloys.

The results of the phase analysis allowed us to identify α-Mg and Mg_2_Ca, Ca_2_Mg_6_Zn_3_, and Mg_26_Zn_59_Gd_7_ intermetallic phases in MgCa5-xZn1Gdx (x = 1, 2, and 3 wt. %) alloys (Figure 1). The α-Mg, Mg_2_Ca, and Ca_2_Mg_6_Zn_3_ phases are in agreement with the phase equilibrium diagram and the following works [2,14,32]. The detected Mg_26_Zn_59_Gd_7_ phases are a part of the finding [44]. It can be observed that a decrease of Ca content in the alloys with Gd addition leads to the formation of a low volume of Mg_2_Ca and Ca_2_Mg_6_Zn_3_ secondary phases. Moreover, the addition of Gd up to 2 wt. % caused only a slight increase of Mg_26_Zn_59_Gd_7_ phases volume. Above this concentration no visible changes in the microstructure were observed.

**Figure 2:**
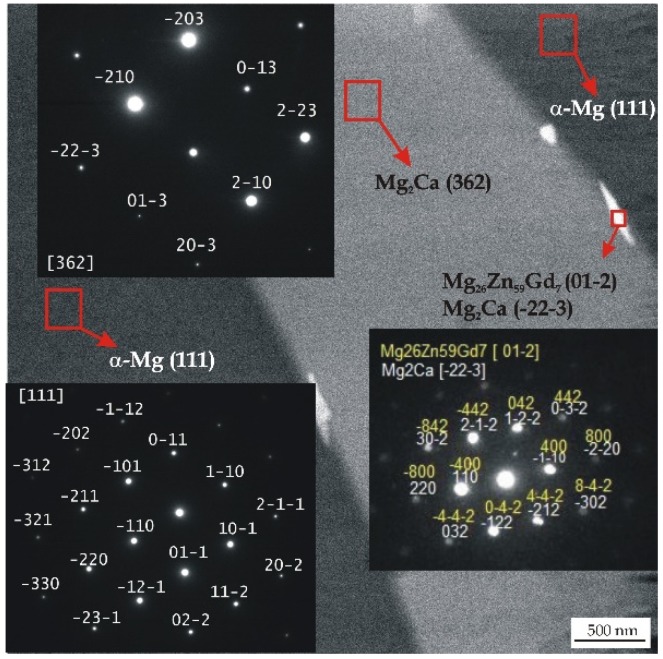
TEM micrograph and corresponding selected area electron diffraction (SAED) patterns from selected areas of the MgCa2Zn1Gd3 alloy.

All detected Mg_26_Zn_59_Gd_7_, Ca_2_Mg_6_Zn_3_, and Mg_2_Ca phases with a hexagonal microstructure belong to the same space group of P63/mmc. The Gd-containing compounds have following lattice parameters: a = 14.633 Å, b = 14.633 Å, and c = 8.761 Å [44]. These lamellar phases with lengths of about 100 nm are distributed along the interdendritic regions (Figure 2).

**Figure 3:**
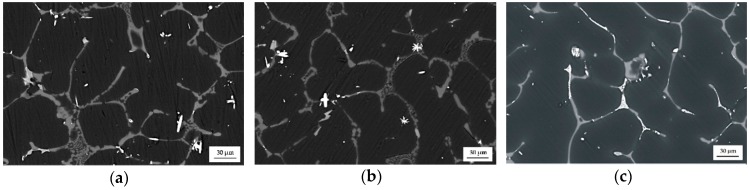
SEM images of: (**a**) MgCa4Zn1Gd1; (**b**) MgCa3Zn1Gd2; and (**c**) MgCa2Zn1Gd3 alloys.

**Figure 4:**
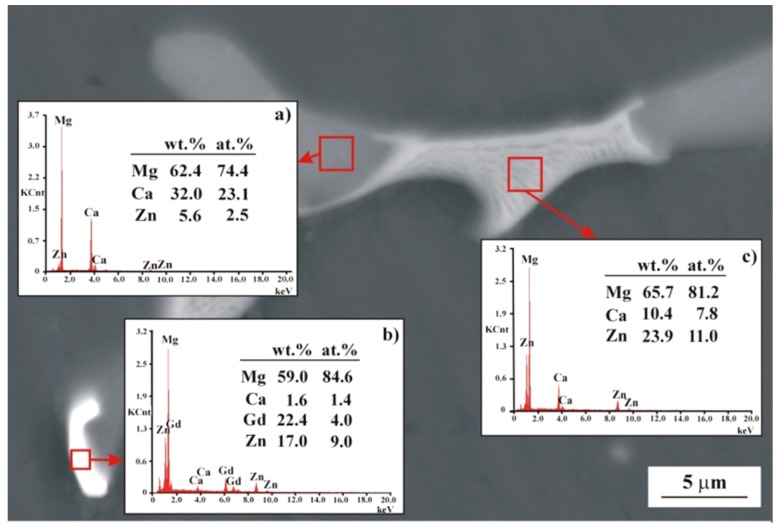
SEM image of MgCa3Zn1Gd2 alloy with EDS spectra (**a**–**c**) from selected areas.

The as-cast MgCa5-xZn1Gdx (x = 1, 2, and 3 wt. %) alloys show a dendritic microstructure with interdendritic solute rich regions. The microstructure of the studied alloys is composed of a primary α-Mg phase and (α-Mg + Mg_2_Ca), (α-Mg + Mg_2_Ca + Ca_2_Mg_6_Zn_3_), and (α-Mg + Mg_26_Zn_59_Gd_7_) eutectics that are distributed at grain boundaries (Figure 3). It can be observed that the eutectics volume decreases with the addition of gadolinium. This is because of the low volume of Mg_2_Ca and Ca_2_Mg_6_Zn_3_ intermetallic phases that fill more space among the grain boundaries. Moreover, it can be observed that Gd-containing phases distributed along interdendritic regions appear as bright areas (Figure 2 and Figure 4) [30].

The microstructure of Mg-based alloy with 22 wt. % Gd addition was observed by Vlcek et al. [20]. After microscopic observations they confirmed that the Mg22Gd alloy exhibits a dendritic microstructure with Mg_46_Gd_9_ intermetallic phase formed in grain boundaries [12]. Shi et al. [14] examined a microstructure of Mg-10Gd-xCa-0.5Zr (x = 0, 0.3, and 1.2 wt. %) alloys and stated that the Mg_5_Gd phase in the form of bright particles in the α-Mg matrix was also detected.

The SEM images and corresponding EDS spectra (Figure 4) of the MgCa3Zn1Gd2 alloy indicated a formation of the eutectic area (interdendritic region). The region that consists of Mg and the highest Ca than Zn concentration is related to Mg_2_Ca phase (Figure 4a). The area composed of Mg, Ca, Zn, and Gd indicated the Mg_26_Zn_59_Gd_7_ phases’ formation (Figure 4b). Moreover, the last region where Mg, Ca, and Zn elements were detected with EDS method belongs to Mg_2_Ca and Ca_2_Mg_6_Zn_3_ intermetallic phases (Figure 4c).

**Figure 5:**
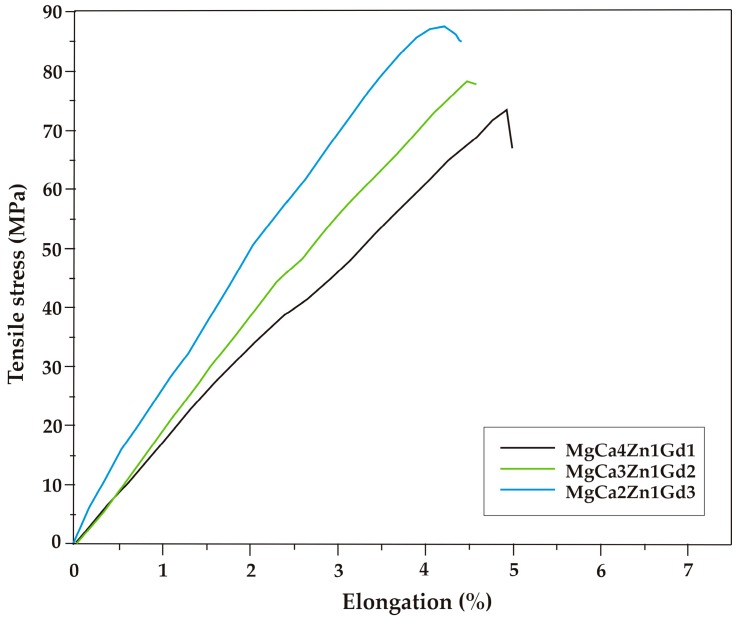
Tensile stress-elongation curves for the Mg alloys with Gd addition.

**Figure 6:**
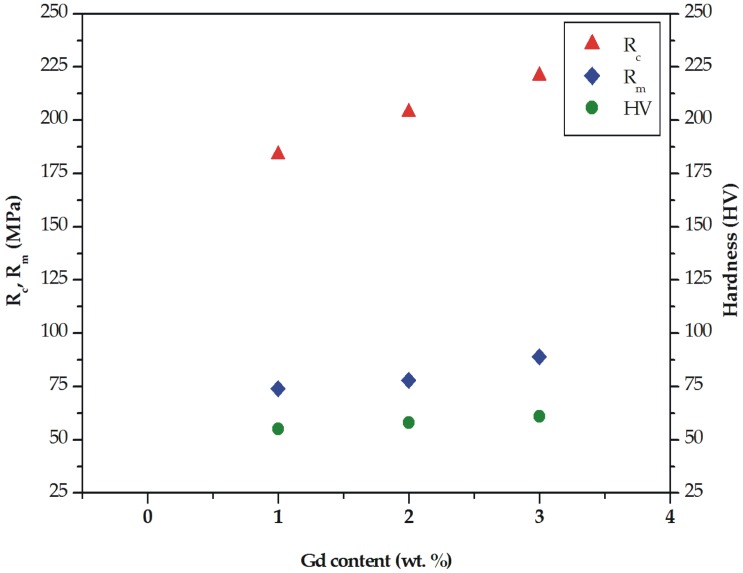
Ultimate tensile strength (R_m_), compressive strength (R_c_), and Vickers hardness for MgCa5-xZn1Gdx (x = 1, 2, and 3 wt. %) alloys.

The results of mechanical tests indicated that tensile properties are improved with gadolinium addition. The selected tensile curves of the studied alloys are shown in Figure 5. The differences in the mechanical behavior can be observed. The improvement of tensile properties as a function of gadolinium addition is due to the solid solution strengthening and precipitation hardening caused by high solubility of Gd in Mg at eutectic temperatures [17,34]. The tensile properties of MgCa5-xZn1Gdx (x = 1, 2, and 3 wt. %) alloys at room temperature are also summarized in Table 1. The ultimate tensile strength (R_m_) equal to 89 MPa (±3.6 SD; ±6.8 CI) was obtained for the MgCa2Zn1Gd3 alloy. The R_m_ of the MgCa4Zn1Gd1 and MgCa3Zn1Gd2 alloys are 74 MPa (±3.0 SD; ±5.7 CI) and 78 MPa (±1.9 SD; ±3.5 CI), respectively (Figure 6).

The addition of gadolinium to the Mg alloys leads to an enhancement of yield strength. The values of tensile yield strength (YTS) increased from 40 to 48 MPa. These results were confirmed by mechanical investigations reported by Zheng et al. [15], Zheng et al. [17] and Gao et al. [34]. The values of tensile yield strength are comparable to YTS obtained for Mg-4Zn-2Gd-1Ca alloy by Wen et al. [36]. The tensile yield strength reached a value of 44 MPa for the studied alloy. Moreover, it can be observed that the addition of gadolinium to the Mg alloys causes a decrease of the elongation (from 4.8% to 4.2%). The improvement of the ultimate tensile strength and a decrease of the elongation with an increase of Gd content in the Mg alloys were observed by Zheng et al. [17] and Li et al. [45], respectively.

The obtained results indicated that the addition of Gd into Mg-based alloys and the formation of Gd-containing phases caused the enhancement of strength (ultimate and tensile yield strength). A slight improvement of tensile properties of the alloys with 2 and 3 wt. % Gd was an effect of a low volume of hard Mg_2_Ca and Ca_2_Mg_6_Zn_3_ intermetallic phases formed along the grain boundaries. They caused embrittlement of Mg alloys [46]. Much higher tensile values compared with the studied Mg-Ca-Zn-Gd alloys could be obtained after heat treatment and plastic forming. Rare earth metals can improve the mechanical properties, high temperature strength and creep resistance of Mg-based alloys [13,14,15,18,30,31,32]. Liu et al. [31] examined the Mg1.5Zn0.2Gd alloy, which was rolled at 450 °C and annealed at 350 °C. They stated that tensile strength of the alloy was above 200 MPa [31].

**Figure 7:**
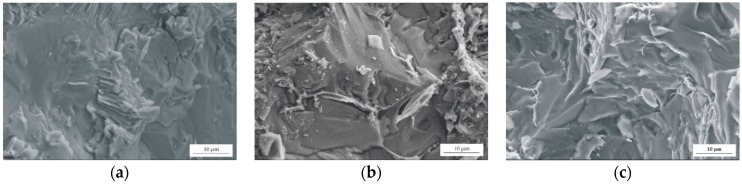
SEM images of selected regions of the fracture surface for: (**a**) MgCa4Zn1Gd1; (**b**) MgCa3Zn1Gd2; and (**c**) MgCa2Zn1Gd3.

The results of tensile tests were complemented by the fracture morphologies of the studied Mg alloys after tensile failure (Figure 7). The fractures of the alloys manifested ductile and fragile behaviors. Some sharp edges, cleavages, areas of shallow dimples, and fatigue cracks (with lengths up to 130 µm) could be seen in the SEM images. The fractures were also characterized with intergranular fracture along the grain boundaries. This is a reason of a decrease in elongation with the increase of Gd content.

The fracture morphologies of Gd-containing alloys were in agreement with the fracture morphologies obtained for Mg-4Zn-2Gd-0.2Ca and Mg-4Zn-2Gd-1Ca alloys. Wen et al. [36] stated that the fracture surface of the Mg-4Zn-2Gd-1Ca alloy is also characterized by intergranular fracture. They noticed that the precipitated phases are mainly distributed in interdendritic regions [36].

The improvement of mechanical properties was also observed for the compressive results. The compressive strength (R_c_) of the studied alloys was 184 MPa (±3.0 SD; ±5.7 CI), 204 MPa (±3.8 SD; ±7.1 CI), and 221 MPa (±4.1 SD; ±7.8 CI) for the Mg alloys with 1, 2, and 3 wt. % Gd, respectively (Figure 6). The compressive parameters at room temperature of MgCa5-xZn1Gdx (x= 1, 2, and 3 wt. %) alloys are listed in Table 2. 

It can be noticed that the addition of Gd into Mg alloys has an effect on the increase of compressive values (compressive yield strength increased from 120 to 135 MPa and compressive strain was improved from 7% to 9%). Improved strength properties of the studied alloys with 2 and 3 wt. % Gd are related to the improvement of microstructure by a high volume of Mg_26_Zn_59_Gd_7_ phases distributed along grain boundaries and a low volume of brittle Mg_2_Ca phases. High compression parameters were obtained for Mg_94_Zn_3_Y_1.5_Gd_1.5_ alloy. Shi et al. [39] stated that compressive stress, compressive yield stress, and compression ratio of this alloy are 414 MPa, 230 MPa, and 18.2%, respectively.

The tensile and compressive properties are complemented by hardness tests. The results of the Vickers hardness indicated that the increase of the gadolinium addition has a slight effect on the hardness changes. The hardness of the studied alloys was improved from 55 HV1 (±2.9 SD; ±2.5 CI) for the alloy with 1 wt. % Gd content to 61 HV1 (±5.6 SD; ±4.9 CI) for the alloy with 3 wt. % Gd. The hardness of the MgCa3Zn1Gd2 alloy was 58 HV1 (±4.4 SD; ±3.8 CI; Figure 6). It could be assumed that the increase of hardness obtained for the alloy with the highest Gd content was caused by a decrease of eutectic volume. The similar hardness, equaled 59 HV, was obtained for Mg-Gd-2Nd-0.5Zr alloy in an as-cast state [15].

The slight improvement of mechanical properties is not enough to select the magnesium alloys for medical application. An important issue is the corrosion resistance of Mg alloys that are very active in a physiological environment [10,11,14,39,46]. Many methods can be used to measure corrosion rates of the Mg alloys. The corrosion rate can be evaluated from the evolved H_2_ volume, Tafel’s extrapolation of polarization curves and electrochemical impedance spectroscopy (EIS) [26]. In the present study the corrosion parameters (e.g., corrosion rates) were measured using Tafel’s extrapolation of polarization curves and the evolved hydrogen volume.

The measurement of the open circuit potential (E_OCP_) changes was used to evaluate the protective properties of layers, which are formed on the surface of alloys exposed to the corrosive environment. It should be noticed that the oxide layer formed on the surface of Mg-based alloys is leaky without a protective barrier. The tightness of oxide layers describes the Pilling–Bedworth ratio, which for Mg alloys is less than 1. There are two types of surface films formed on the Mg during corrosion. In chloride solutions a thin layer of MgO is covered by a loose layer of Mg(OH)_2_ [19]. Both layers exhibit limited protective behaviors. The film formed during preparation of samples is degraded in an initial stage of immersion. At the same time, the second film is formed on the corroding surface of magnesium. To understand the corrosion mechanism of surface layers, X-ray photoelectron spectroscopy (XPS) is often used. This is a technique for a quantitative analysis of chemical changes that occur on the surface of Mg alloys [29]. Finally, it should be noted that the pH of the solution dependent on corrosion is related to the surface films that are formed on Mg in various solutions [19,20,23,24,27,28,47]. The pH increases rapidly in the first hours of immersion to a pH of ~10.3 due to a low solubility of Mg(OH)_2_. This is a significant issue in a corrosion study of Mg alloys for medical application [27]. Human blood has a pH of ~7.4. It is mainly regulated by the lungs through respiration influencing the rate of CO_2_ removal from the blood. The best practice in studying Mg corrosion is to use a large solution volume and take the same CO_2_-bicarbonate buffer as in the body [27,28].

**Figure 8:**
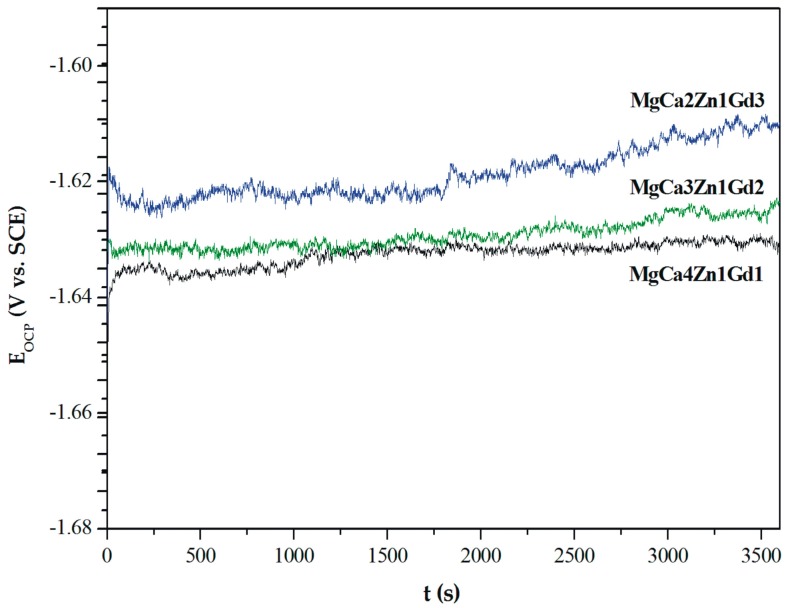
The open circuit potential (E_OCP_) changes as a function of time for studied MgCa5-xZn1Gdx (x = 1, 2, and 3 wt. %) alloys in Ringer’s solution at 37 °C.

The curves determined for a stationary potential as a function of time indicate that all Mg alloys are active in a corrosive environment. However, the MgCa2Zn1Gd3 alloy shows a higher activity in Ringer’s solution than the other tested alloys (Figure 8).

**Figure 9:**
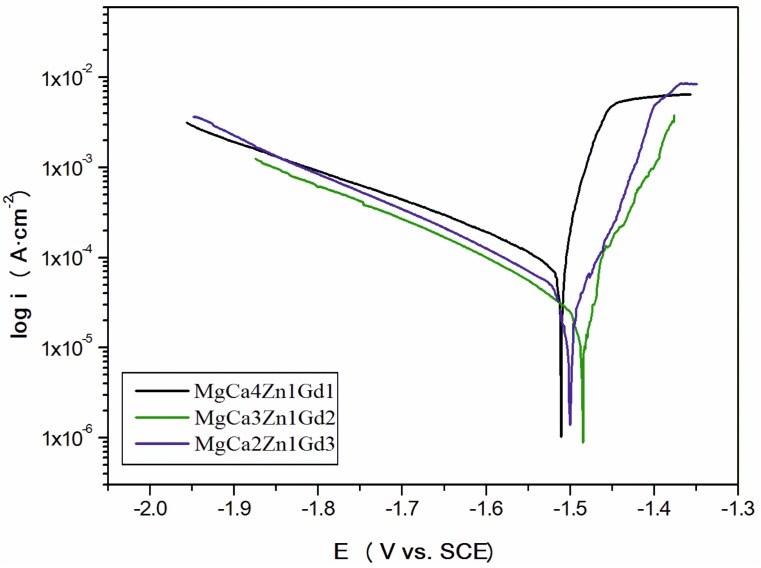
Polarization curves for MgCa5-xZn1Gdx (x = 1, 2, and 3 wt. %) alloys in Ringer’s solution at 37 °C.

The values of E_OCP_ potential are related to a composition of the studied alloys. The increase of gadolinium content causes a slight increase of the open circuit potential. The small differences of the E_OCP_ potential among Gd-containing alloys are from minor changes of the chemical composition. These changes are not higher than 40 mV after 3600 s. Moreover, it can be observed that cathodic polarization shifts the corrosion potential (E_corr_) towards positive values (Figure 9).

The increase of the corrosion potential (about 150 mV for the MgCa3Zn1Gd2**;** E_OCP_ for the alloy was about −630 mV and E_corr_ was −1480 mV), suggests that the studied alloys exhibited quite good corrosion resistance. These changes resulted from the reduction of gadolinium oxides.

Extrapolation of polarization curves using Tafel’s method allowed us to determine the polarization resistance (R_p_) and corrosion current density (i_corr_) of the studied alloys (Table 3). 

The polarization curves provided some useful information to recognize the Mg corrosion mechanism. It can be also noticed that for Mg alloys, Tafel’s extrapolation of polarization curves gives corrosion rate values that typically do not agree with weight loss or hydrogen evolution [19,22,23,27]. In many reports [22,23] we can find that Tafel’s extrapolation can be used to measure an initial corrosion behavior that does not correlate with a real, steady state of corrosion behavior. The lowest value of i_corr_, equaled 51 μA·cm^−2^, and the highest polarization resistance, R_p_, equaled 532 Ω·cm^2^, were observed for the MgCa3Zn1Gd2 alloy. A good corrosion resistance of this alloy confirmed a value of corrosion rate, v_corr_, which equals 0.62 mm·y^−1^. A significant deterioration of the corrosion resistance indicates the alloy with 1 wt. % Gd content. The corrosion current density for this alloy was higher and it was 170 μA·cm^−2^ (R_p_ was 110 Ω·cm^2^). MgCa4Zn1Gd1 was characterized by the highest corrosion rate (v_corr_ was 1.86 mm·y^−1^). This is because of a high Ca content. A high volume of Mg_2_Ca phases significantly accelerates anodic kinetics of magnesium [39]. A further increase of the gadolinium content caused a slight increase of corrosion current density (i_corr_ for the alloy with 3 wt. % Gd was 98 μA·cm^−2^). The corrosion rate of the MgCa2Zn1Gd3 alloy was 1.48 mm·y^−1^.

Srinivasan et al. [18] studied the corrosion behaviors of two Mg-10Gd-2Zn and Mg-10Gd-6Zn alloys in 0.5 wt. % NaCl solution. They compared the corrosion parameters after different immersion times, for 0.5, 24, and 100 h. The corrosion potentials obtained for these alloys and immersion times are similar to the E_corr_ values measured for the studied alloys. The corrosion potentials for Mg-10Gd-2Zn are −1.551 V, −1.480 V, and −1.505 V after 0.5, 24, and 100 h of immersion, consequently. While, E_corr_ for the Mg-10Gd-6Zn alloy are −1.495 V, −1.480 V, and −1.498 V after the same immersion times. Moreover, the best values of corrosion current densities were obtained (e.g., i_corr_ for Mg-10Gd-6Zn is 65 μA·cm^−2^ after 0.5 h of immersion, 128 μA·cm^−2^ after 24 h of immersion and 193 μA·cm^−2^ after 100 h of immersion).

**Figure 10:**
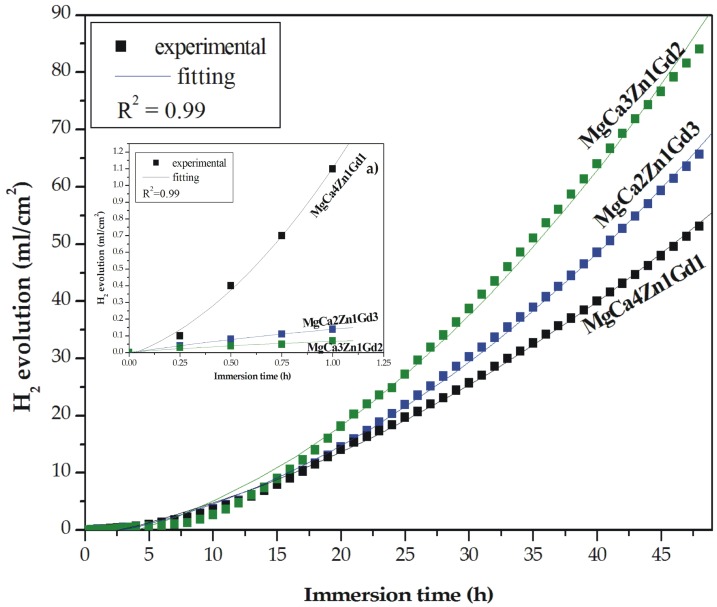
Hydrogen evolution volume as a function of immersion time in Ringer’s solution at 37 °C for studied MgCa5-xZn1Gdx (x = 1, 2, and 3 wt. %) alloys during 48 h and during 1 h (a).

Hydrogen evolution is an important issue of magnesium corrosion mechanism [20,22,23]. A hydrogen evolution method is an easy and reliable method for measuring and monitoring the corrosion rate of Mg-based alloys. It was firstly described by Song et al. [48]. One of the advantages of this method is a high experimental efficiency with smaller theoretical and experimental errors. The results of immersion tests for the MgCa4Zn1Gd1, MgCa3Zn1Gd2, and MgCa2Zn1Gd3 alloys are presented as the hydrogen evolution volume during 48 h of immersion (Figure 10). The corrosion products were formed slowly on the surface during the first hour of immersion; the degradation rate of the alloys with 2 and 3 wt. % Gd was slow (Table 4). We could assume that after 1 h of immersion of the studied alloys in the artificial environment the corrosion rates calculated from the hydrogen evolution volume were comparable to the results of polarization data (Table 3). 

In the case of the MgCa4Zn1Gd1 alloy the corrosion rate, v_corr_, reached the highest value of 0.16 mm·y^−1^ (v_corr_ based on the i_corr_ value was 1.86 mm·y^−1^); the volume of evolved H_2_ was 1.1 mL·cm^−2^·h^−1^. MgCa3Zn1Gd2 alloy was characterized by the lowest corrosion rate, which was 0.10 mm·y^−1^ (evolved H_2_ volume was 0.07 mL·cm^−2^·h^−1^) after 1 h of immersion and the v_corr_ from polarization data was 0.62 mm·y^−1^. Similar correlation between corrosion rates and the Gd content in the Mg alloys was observed after 8 h of immersion. The v_corr_ of the alloys with 1 and 3 wt. % Gd were 1.14 and 1.67 mm·y^−1^, consequently (Table 4).

After 48 h of immersion a high dissolution of the Mg alloys was observed. The H_2_ volume increased significantly and reached values of 53.11, 84.11, and 65.69 mL·cm^−2^ for the MgCa4Zn1Gd1, MgCa3Zn1Gd2, and MgCa2Zn1Gd3 alloys, respectively (Figure 10). The corrosion rate of the alloy with 1 wt. % Gd content was 5.97 mm·y^−1^ and this is an effect of Ca addition and a high volume of Mg_2_Ca secondary phases formation. These intermetallic phases were distributed along grain boundaries and caused grain refinement. The decrease of the grain size leads to the formation of a more dense passive layer, on which the corrosion products are an effective barrier against corrosion process [36]. Two other studied alloys are characterized by an increase of corrosion rates; v_corr_ were 6.63 and 8.22 mm·y^−1^ for the MgCa2Zn1Gd3 and MgCa3Zn1Gd2 alloys, consequently. Zhang et al. [32] measured a corrosion rate of Mg-9Gd-1Zn-0.6Ca alloy. They reported, similarly as in the case of our investigations, that the tested alloy has a higher corrosion resistance among two other alloys with 0.2 wt. % Ca and without Ca content.

The decrease of long term corrosion properties was also noticed by Srinivasan et al. [13]. They carried out the immersion tests in 0.5 wt. % NaCl solution during 400 h for Mg-2Gd-2Zn, Mg-2Gd-6Zn, Mg-10Gd-2Zn, and Mg-10Gd-6Zn alloys. It was confirmed that an increase of H_2_ volume for the alloy with 2 wt. % Gd is much lower than that for the alloy with 10 wt. % Gd content. The ternary Mg-10Gd-1.2Ca-0.5Zr alloy in as-cast state examined for 14 days in cell culture medium (CCM) at 37 °C by Shi et al. [14] is also characterized by a fast corrosion rate. The investigation results of an impact of Mg-Gd alloys on a long-term corrosion behavior in 1 wt. % NaCl were presented by Hort et al. [49]. They stated that corrosion rates of alloys with Gd addition are reduced until the gadolinium occurs in solid solution.

**Figure 11:**
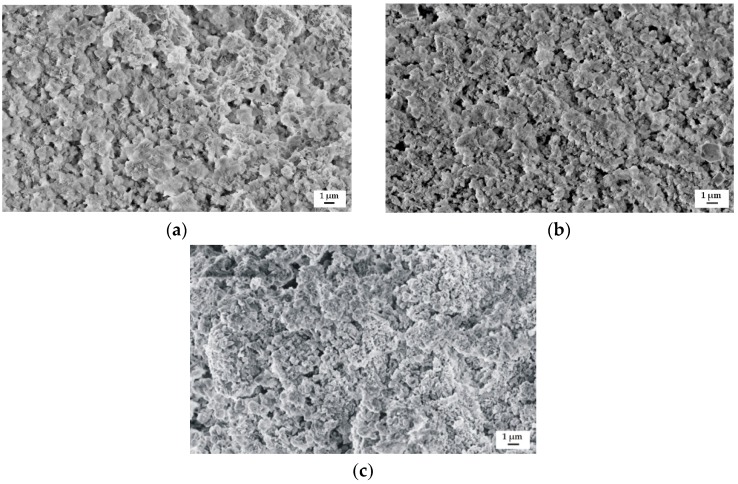
SEM images of samples’ surface with corrosion products of: (**a**) MgCa4Zn1Gd1; (**b**) MgCa3Zn1Gd2; and (**c**) MgCa2Zn1Gd3 alloys after immersion tests in Ringer’s solution.

The samples surface morphology of the studied alloys after immersion in Ringer’s solution for 48 h is presented in Figure 11. The corrosion products on the surfaces of all Gd-containing alloys were visible. The corroded surface of the MgCa4Zn1Gd1 alloy contained a more dense concentration of corrosion products (Figure 11a). It is in agreement with the long-period hydrogen evolution data.

**Figure 12:**
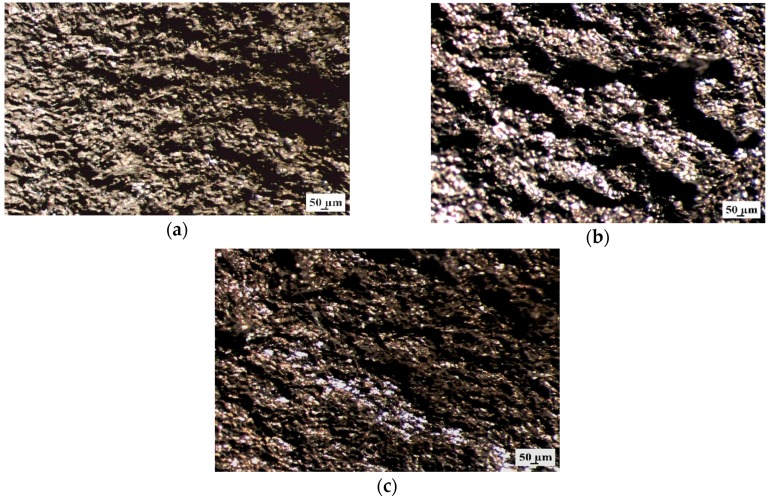
Stereoscopic images of samples’ surface without corrosion products of: (**a**) MgCa4Zn1Gd1; (**b**) MgCa3Zn1Gd2; and (**c**) MgCa2Zn1Gd3 alloys after immersion tests in Ringer’s solution.

Additionally, the samples surface without corrosion products after 48 h of immersion was observed using stereoscopic microscope (Figure 12). It can be seen that all Mg alloys with gadolinium showed the pitting corrosion behaviors. However, the MgCa4Zn1Gd1 and MgCa2Zn1Gd3 alloys exhibited smaller corrosion damages.

The similar corrosion mechanism was also stated by Song et al. [21]. Atrens et al. [19] explained that in chloride solutions (e.g., Ringer’s solution) the corrosion appears at breaks in the steady state surface films (consisting of MgO and Mg(OH)_2_ films [21]) and chloride ions contribute to form deeper pits.

Based on the experimental results, it could be concluded that MgCa5-xZn1Gdx (x = 1, 2, and 3 wt. %) alloys are very interesting and promising biomaterials for potential applications in the human body. Nevertheless, the alloys were characterized by high hydrogen evolution volume, so it is important in the selection of materials for medical implants (H_2_ volume tolerated by human body without hazardous effects for the health is 0.01 mL·cm^−2^ per day [50]). The authors intend to improve the long period corrosion results by using protective coatings (e.g., MgO, and ZnO) or plastic forming.

## 4. Conclusions

1. In the present study the mechanical and corrosion properties of a new designed MgCa5-xZn1Gdx (x = 1, 2, and 3 wt. %) alloys were presented.
2. The increase of Gd content in Mg alloys had an effect on the microstructure changes. A small reduction of eutectic volume by a low volume of Mg_2_Ca and Ca_2_Mg_6_Zn_3_ secondary phases could be observed.
3. The addition of Gd in the studied alloys resulted in the improvement of ultimate tensile strength (R_m_ for the alloys with 1, 2, and 3 wt. % Gd were 74, 78, and 89 MPa, respectively) and a decrease of the elongation (from 4.8% to 4.2%).
4. The increase of Gd content also improved the compressive properties (for example, the compressive strength, R_c_*,* were 184, 204, and 221 MPa for the MgCa4Zn1Gd1, MgCa3Zn1Gd2, and MgCa2Zn1Gd3 alloys, consequently).
5. The Vickers hardness increased from 55 to 61 HV1 for the alloys with 1–3 wt. % Gd.
6. The results of electrochemical corrosion tests showed that the studied alloys were characterized by good corrosion resistance. The corrosion potential (E_corr_) of the alloys shifted towards positive values in comparison to the E_OCP_ potential.
7. The electrochemical corrosion rates corresponded with the corrosion rates measured in an aggressive environment during 8 h with immersion tests. The high corrosion resistance estimated by the evolved H_2_ volume after 1 and 8 h of immersion was indicated for the alloy with 2 wt. % Gd. The v_corr_ were 0.10 and 1.14 mm·y^−1^ for 1 and 8 h, respectively. The corrosion rates changed with time and after 48 h of immersion, the lowest v_corr_ equaled 5.97 mm·y^−1^ was noticed for the MgCa4Zn1Gd1 alloy.

## Figures and Tables

**Table 1:** Values of static tensile tests of MgCa5-xZn1Gdx (x = 1, 2, and 3 wt. %) alloys.

Alloy	Tensile Yield Strength, YTS, MPa	Ultimate Tensile Strength, R_m_, MPa	Maximum Elongation, A_t_, %
MgCa4Zn1Gd1	40 ± 1.7	74	4.8
MgCa3Zn1Gd2	42 ± 2.4	78	4.4
MgCa2Zn1Gd3	48 ± 2.9	89	4.2

**Table 2:** Values of static compressive tests of MgCa5-xZn1Gdx (x = 1, 2, and 3 wt. %) alloys.

Alloy	Compressive Yield Strength, YCS, MPa	Compressive Strength, R_c_, MPa	Compressive Strain, %
MgCa4Zn1Gd1	120 ± 4.6	184	7.0 ± 1.1
MgCa3Zn1Gd2	130 ± 3.9	204	8.5 ± 0.9
MgCa2Zn1Gd3	135 ± 5.1	221	9.0 ± 1.3

**Table 3:** Corrosion parameters of MgCa5-xZn1Gdx (x = 1, 2, and 3 wt. %) alloys.

Alloy	Corrosion Potential, E_corr_, V	Polarization Resistance, R_p_, Ω·cm^2^	Corrosion Current Density, i_corr_, μA·cm^−2^	Corrosion Rate, v_corr_, mm·y^−1^
MgCa4Zn1Gd1	−1.51 ± 0.03	110 ± 2	170 ± 7	1.86 ± 0.02
MgCa3Zn1Gd2	−1.48 ± 0.03	532 ± 10	51 ± 2	0.62 ± 0.02
MgCa2Zn1Gd3	−1.50 ± 0.03	480 ± 9	98 ± 4	1.48 ± 0.02

**Table 4:** Corrosion rates calculated from the hydrogen evolution volume after 1, 8, and 48 h of immersion for MgCa5-xZn1Gdx (x = 1, 2, and 3 wt. %) alloys.

Alloy	Corrosion Rate, v_corr_, mm·y^−1^ (after 1 h)	Corrosion Rate, v_corr_, mm·y^−1^ (after 8 h)	Corrosion Rate, v_corr_, mm·y^−1^ (after 48 h)
MgCa4Zn1Gd1	0.16 ± 0.03	2.66 ± 0.02	5.97 ± 0.02
MgCa3Zn1Gd2	0.10 ± 0.03	1.14 ± 0.02	8.22 ± 0.02
MgCa2Zn1Gd3	0.11 ± 0.03	1.67 ± 0.02	6.63 ± 0.02
